# Struggling over new asset geographies

**DOI:** 10.1177/20438206231189580

**Published:** 2023-07-25

**Authors:** Kean Birch, Callum Ward

**Affiliations:** 7991 York University, Canada; 8097 Uppsala University, Sweden

**Keywords:** Assetization, capitalization, financialization, valuation, value

## Abstract

In this response, we address criticisms of our definition of assetization from an accounting perspective, its overlap with financialization, and the relationship between value and valuation it posits. We reflect on a future agenda around assetization emphasizing the political dimensions of externalizing future costs and the implications of rising inflation.

## Introduction

In ‘Assetization and the New Asset Geographies’ (Birch and Ward, 2024), we overviewed the burgeoning human geography literature on the transformation of things into (financial) assets. We argued that these heterogeneous empirical accounts point to a common focus on the moments and mechanisms of enclosure, extraction, and capitalization necessary for the socio-technical achievement of the asset form.

Our argument was that ‘assetization’ – the process of transforming different things into the asset form – is a common problematic requiring conceptual borrowing and sharpening of theoretical boundaries across theoretical approaches. To this end, we are grateful for the four incisive, constructive responses pushing us for such sharpening from the perspective of critical accounting (Chiapello, 2024), international political economy (Purcell, 2024), and economic geography (Strauss, 2024; Ouma, 2024). Here we briefly respond to their comments and implications for an agenda around assetization.

## Making the asset form

Eve Chiapello points out that we conflate two distinct meanings of ‘asset’: first, in accounting, an asset is a resource ‘available for business operations’ listed on a company's balance sheet; and second, in asset management, an asset is a financial instrument representing a particular ‘risk-return’. She argues that our use of the term assetization leaves out the accounting definition of assets, especially as this relates to intangible assets. Consequently, our focus on mobilizing things as financial assets more generally, Chiapello argues, overlaps too much with the concept of financialization.

This provides necessary definitional nuance between non-financial assets and financial assets, but we do not believe that this important distinction amounts to the binary Chiapello seems to suggest. A major component of the empirical puzzle which assetization addresses is that the distinction between a balance sheet asset and a financial asset has blurred as a result of the changes to fair value accounting, which Chiapello outlines. In this context, the demarcation of balance sheet assets (directly or indirectly) can still produce a capitalizable object *qua* a (quasi)-financial asset (see, e.g. Bryan et al., 2017’s discussion of the treatment of IP as financial assets within wealth chains). Unlike Chiapello, however, we view the drawing of an asset boundary between the balance sheet and financial instruments not as a discrete act but as a crucial moment in a wider set of processes of revenue generation, capitalization, *and* social and geographical formation.

A financial asset is distinguished by the existence of a counterparty who has a commensurate obligation (i.e. liability), while a non-financial asset does not. Assetization helps us to identify a broader set of counter-parties to all sorts of assets; that is, social actors who have financial *and* non-financial liabilities attached to them through the techno-economic configuration of something as an asset. For example, asset management practices, which are increasingly being implemented by governments, can end up locking-in (future) citizens to bearing particular costs which have been temporally displaced through new accounting and calculative practices. Rather than adhering to a separation between the boundary making of balance sheet assets and the market making of financial risk-return, then, assetization highlights the interaction of both and their shaping of the logics and modes of financialized understanding, resource extraction, governance, and associated geographical restructuring.

Finally, there is certainly an overlap with assetization as we use it and financialization, but this speaks primarily to the conceptual overextension of financialization. Financialization denotes a relative relationship to finance in highlighting a particular direction towards more dominance of financial actors, their practices, and narratives (Aalbers, 2019). However, as a concept financialization does not necessarily imply attention to the creation of assets, and does not speak to the sociotechnical processes of enclosure and rent extraction implicated in the making of an asset. This overextension both obscures these important empirical processes and blunts the power of financialization as an explanatory concept relating to the increasing dominance of finance and financial practices.

## Reifying future values

An important part of our argument is that assetization offers a common problematic and concept to bridge critical political economy analyses of value extraction and the focus in social studies of finance on valuation. In a rigorous engagement from a value-theoretical perspective, Thomas Purcell expresses significant reservations about this approach encapsulated in his rhetorical question: ‘common problems or different questions?’ He argues that our conceptualization of assetization is weighted towards constructivist ontologies because it trains attention on the subjective process of valuation, so untethering analysis from ‘an objective substance like “value”’. To this, we note that asking different questions of a common problem is the essence of a pluralistic agenda, but such pluralism depends on acknowledging that the answers produced by one set of questions may overlook important aspects of the phenomena which another offers insight on.

Purcell spurns analyses which emphasize the future-orientated nature of valuation on the basis that assets are ‘a form of appearance of value’, which ‘… correspond to the amount of “capital” that would yield the given amount of income at the prevailing rate of interest’. The unacknowledged blindspot here is that of how social actors arrive at and materialize what amount of capital ‘*would*’ (a future-orientated hypothetical) yield this ‘given’ amount of income. Only by taking these valuation questions as given can asset prices then be treated as mechanistically tethered to value in production. In practice, financiers do not have insight into the unfetishized substance of value and so engage in systemic speculation in their price setting which takes on its own (quasi-)autonomous dynamics (see Ward, 2021 for an account of this within a theorization of the circuits of capital).

The core feature of fictitious capital is its crystallization of posited future value into present circulation in a way that has material force. In our emphasis on real abstraction to capture this dynamic we insist that analysis must grasp the interplay of subjective assessment becoming the fetishized object itself through the process of reification. Ignoring the material force of fictitious capital formation in contemporary capital circuits is not a materialist alternative unless one simply equates materialism with productivism. Rather than eliding class and production, theorizing valuation as an active moment in capital circulation is necessary if we are to unpack the very material processes (re-)shaping the geographies of value chains and societal struggles over surplus today.

## Politicizing the asset form

Purcell's commentary encapsulates a concern running throughout the four responses that our magnification of the role of economic rents and enclosure in new asset geographies risks overlooking the enduring importance of competition, commodity and value chains, and labor markets. Strauss (2024) and Ouma's (2024) commentaries centre on this concern, but do not locate the problem in a fundamental ontological or definitional difference. Rather, they broadly accept the problematic of assetization while seeking to address this concern by extending the scope of analysis beyond the limitations of our initial account.

Ouma argues that the asset form is very much part of the ‘economic DNA’ of contemporary capitalism and stresses the need for methodological tools to unpack the conflicts and distributional struggles underlying assetization in a ‘visual politics of the asset form’. Ouma links this to the work of externalization that characterized imperialism and what he terms the global return society, in the process pointing to a deeper and longer history of assets than that which we presented. Within this, he highlights the need to not lose sight of the importance of commodity production alongside asset construction, a point we very much endorse in the need to understand the recursive intersections of value and wealth chains.

Strauss foregrounds the importance of institutionalized accumulation by dispossession in the enclosure of assets and their impact on social reproduction. She highlights how such enclosure is predicated on the ‘restructuring and devaluation of labor’ and points to how dialogue across social studies of finance and political economy is necessary to understand the violence of this process. Rightly insisting on the intrinsic role of social reproduction and (paid and unpaid) labour precarity in assetization, Strauss applies this to Canada's housing-based ‘asset economy’ (see Adkins et al., 2020) and the uneven impacts of recent inflation within such a social configuration.

With this emphasis on inflation, Strauss has possibly identified the most urgent direction for an agenda on assetization. Following supply chain shocks, recent inflation represents a significant disruption to the asset-based socioeconomic settlements which arose in the context of low-interest rates and cheap money. There is limited evidence that this is being driven by the so-called Keynesian ratchet of rising wages begetting rising prices in an upward spiral (Birch, 2017), as wage inflation has not preceded or matched price rises (Weber et al., 2022). Rather, we are in a situation of profit-driven inflation compounded, as Strauss points out, by wage-focused policy and political responses, like pushing up interest rates, which disproportionately impacts the poorest members of society. Here, the asset structure of costs and risks that is central to the politics of the asset society (see Adkins et al., 2020) are being disrupted by inflation, but these disruptions are playing out in unpredictable and explicitly contested ways with uneven impacts across racialized, gendered, and classed lines.

Profit-driven inflation highlights the extent to which ‘normal’ policy levers has come undone as countries around the Global North have instituted a low-interest rate regime over the last decade. But not only have these policy levers come undone, they have shifted so dramatically precisely because of the political and policy concern with protecting asset ownership. The attendant consequences that reversing this regime are socially, politically, and economically unpalatable to elites. The high value of assets is tied to low-interest rates, especially certain forms of assets like housing, and much of the political economy of ‘northern’ countries has been reconfigured in pursuit of these asset values.

## Conclusion

As a future agenda, then, we need to examine assets in their diverse forms in order to understand how to avoid the further entrenching of the uneven distribution and allocation of societal resources. Our paper and the commentaries on it illustrate the need to unpack the manifestation and geographies of the asset form so that we can find the necessary ways to challenge assetization in our societies. A particularly important objective is to work out how to reverse the course of the last decade or so, which will necessitate new analytical and methodological tools to address the distinctly messy capitalism in which we find ourselves.
